# On the Difficulties and Advantages of Catheterism of the Air-Passages in Diseases of the Chest

**Published:** 1860-06

**Authors:** 


On the Difficulties and Advantages of Catheterism of the Air-Passages in Diseases of the Chest. By Horrace Green, M. D., L. L. D., etc. New York.

This is an essay of 24 pages, in which the author endeavors to show that it is practicable to introduce a proper tube into the trachea, and through it into the larger bronchial tubes, and to inject a solution of Nitrate of Silver into the lungs; not only without injurious effects, but with benefit.

That such catheterism and injections are practicable, we are not disposed to deny. But that they are beneficial or even free from danger, is very far from having been proved by the author; and we would advise all practitioners to be very cautious how they attempt to imitate the practice recommended by Dr. H. Green.

